# Timing for the second fecal microbiota transplantation to maintain the long-term benefit from the first treatment for Crohn’s disease

**DOI:** 10.1007/s00253-018-9447-x

**Published:** 2018-10-24

**Authors:** Pan Li, Ting Zhang, Yandong Xiao, Liang Tian, Bota Cui, Guozhong Ji, Yang-Yu Liu, Faming Zhang

**Affiliations:** 1 Medical Center for Digestive Diseases, The Second Affiliated Hospital of Nanjing Medical University, Nanjing, 210011 China; 2 Key Lab of Holistic Integrative Enterology, Nanjing Medical University, Nanjing, 210011 China; 3 Channing Division of Network Medicine, Brigham and Women’s Hospital and Harvard Medical School, Boston, MA 02115 USA; 4 Science and Technology on Information Systems Engineering Laboratory, National University of Defense Technology, Changsha, 410073 China; 5 Department of Physics, Hong Kong Baptist University, Kowloon Tong, Hong Kong SAR China; 6 Center for Cancer Systems Biology, Dana-Farber Cancer Institute, Boston, MA 02115 USA

**Keywords:** Fecal microbiota transplantation, Crohn’s disease, Gut microbiota, Urine metabolomics

## Abstract

Increasing evidence has shown that fecal microbiota transplantation (FMT) could be a promising treatment option for Crohn’s disease (CD). However, the frequency of FMT for CD treatment remains unclear. This study aimed to evaluate the optimal timing for administering the second course of FMT to maintain the long-term clinical effects from the first FMT for patients with CD. Sixty-nine patients with active CD who underwent FMT twice and benefited from the first FMT were enrolled in this study. Clinical response, stool microbiota, and urine metabolome of patients were assessed during the follow-up. The median time of maintaining clinical response to the first FMT in total 69 patients was 125 days (IQR, 82.5–225.5). The time of maintaining clinical response to the second FMT in 56 of 69 patients was 176.5 days (IQR, 98.5–280). The fecal microbiota composition of each patient post the first FMT was closer to that of his/her donor. Compared to that of the baseline, patients prior to the second course of FMT showed significant differences in urinary metabolic profiles characterized by increased indoxyl sulfate, 4-hydroxyphenylacetate, creatinine, dimethylamine, glycylproline, hippurate, and trimethylamine oxide (TMAO). This study demonstrated that patients with CD could be administered the second course of FMT less than 4 months after the first FMT for maintaining the clinical benefits from the first FMT. This was supported by the host–microbial metabolism changes in patients with active CD. **Trial registration**: ClinicalTrials.gov, NCT01793831. Registered 18 February 2013. https://clinicaltrials.gov/ct2/show/NCT01793831?term=NCT01793831&rank=1

**Electronic supplementary material:**

The online version of this article (10.1007/s00253-018-9447-x) contains supplementary material, which is available to authorized users.

## Introduction

Crohn’s disease (CD) is a chronic inflammatory relapsing disorder of the gastrointestinal tract, and growing evidence suggests that dysbiosis of the gut microbiota contributes to pathogenesis (Gevers et al. 2014; Pascal et al. 2017). Previous studies have shown that administration of fecal microbiota transplantation (FMT) could effectively induce clinical response in patients with active CD (Colman and Rubin 2014; Cui et al. 2015a; Goyal et al. 2018; Suskind et al. 2015; Vaughn et al. 2016). However, the patients’ clinical response to single FMT is short lived. Recently, our group reported that sequential FMTs could induce and maintain a sustained clinical remission for patients with active CD complicated with abdominal inflammatory masses (He et al. 2017). However, the frequency of FMT to maintain patients’ long-term clinical efficacy in CD needs further study. We hypothesized that the time of maintaining clinical response after the second course of FMT might be equal or longer than that after the first FMT.

Several clinical trials in inflammatory bowel disease (IBD) have concluded that clinical response to FMT is associated with a post-FMT increase in bacterial diversity (Goyal et al. 2018; Moayyedi et al. 2015; Vaughn et al. 2016). This is related to the fact that donor microbiota can be successfully engrafted and sustained for a variable period of time (Simone et al. 2016). However, very little is known about whether the transfer of a community of highly dynamic and metabolically active microbiota through FMT could regulate the metabolism of patients with CD. The metabolome, which consists of the end products of metabolism with low molecular weight, represents the ultimate response of the body under a certain condition, such as disease and specific treatments. Metabolomics provides a unique strategy for identifying biologically significant metabolic changes that occur in an organism in response to bacteriotherapy (Bazanella et al. 2017; Landy et al. 2015; Miccheli et al. 2015). This prompts us to utilize metabolomics to identify potential microbiota–metabolic signatures to monitor the clinical response to FMT in patients with active CD.

In this article, we described the clinical response to the first two FMTs in CD patients from a prospective study of serial FMTs performed in patients with active CD. Patients who benefited from the first two FMTs were serially followed for adverse effects and clinical response. In addition, gut microbiome and urine metabolome analysis were performed on samples from a subgroup of randomly selected nine recipients at pre- and post-transplant in the first FMT, and their respective donors.

## Materials and methods

### Patients and donors

This study as a part of a clinical trial (NCT01793831) was performed at the Second Affiliated Hospital of Nanjing Medical University, Nanjing, China. This study was reviewed and approved by the institutional ethical committee. Patients were recruited from November 2012 to September 2016, and the last follow-up was completed on April 1, 2017. All eligible subjects provided written informed consents prior to participation in this study.

Eligible patients were aged ≥ 14 years with active CD defined as Harvey–Bradshaw Index (HBI) score > 4 despite treatment with 5-aminosalicylic acid (5-ASA), corticosteroids, immunomodulators, and/or anti-tumor necrosis factor (TNF) agents. All eligible patients received at least the first two FMTs and achieved clinical efficacy from the first FMT. Patients were excluded if they accompanied with other severe diseases, including other intestinal diseases, e.g., *Clostridium difficile* infection, diabetes, cancers, or failed to complete the follow-up.

Patients could have choice to self-identify their potential donors as candidates, such as their family members, relatives, or friends. The most source of donors was from our universal fecal microbiota bank (China fmtBank). Selected donors were screened by strict exclusion criteria, which were described in our previous publications (Cui et al. 2015a, 2015b; He et al. 2017).

### FMT procedure

As previously reported protocol (Cui et al. 2015a, 2015b; He et al. 2017), our original FMT preparation method was termed filtration plus centrifugation (FPC). Subsequently, an automatic purification system (GenFMTer; FMT Medical, Nanjing, China) was used to purify microbiota in our protocol, which was termed as microfiltration plus centrifugation (MPC). Since 2014, the standardized protocol was performed in a Good Manufacturing Practice (GMP)-level laboratory and workflow (Zhang et al. 2018). The general procedures briefly include microfiltration, centrifugation, washing, discarding, and dilution. The fresh stool was collected in a disposable bucket, which was designed for the GenFMTer machine (FMT Medical, Nanjing, China). We adopted the “one-hour FMT protocol” which requires that process time from feces defecation to the fresh bacterial material be infused into the patient’s intestine is within 1 h (He et al. 2017; Zhang et al. 2018).

The fresh microbiota suspension could be infused into the distal duodenum of patients through a gastroscope under anesthesia. In order to prevent the refluxing of microbiota liquid and inhibit the secretion of gastric acid, patients were given metoclopramide 10 mg by intramuscular injection and proton pump inhibitor intravenously at least 1 h before FMT (Cui et al. 2015a). Another way to transplant fecal microbiota into the mid-gut was through the mid-gut/nasal–jejunal transendoscopic enteral tubing (TET) tube (FMT Medical, Nanjing, China) (Long et al. 2018).

### Study design

**Fig. 1:**
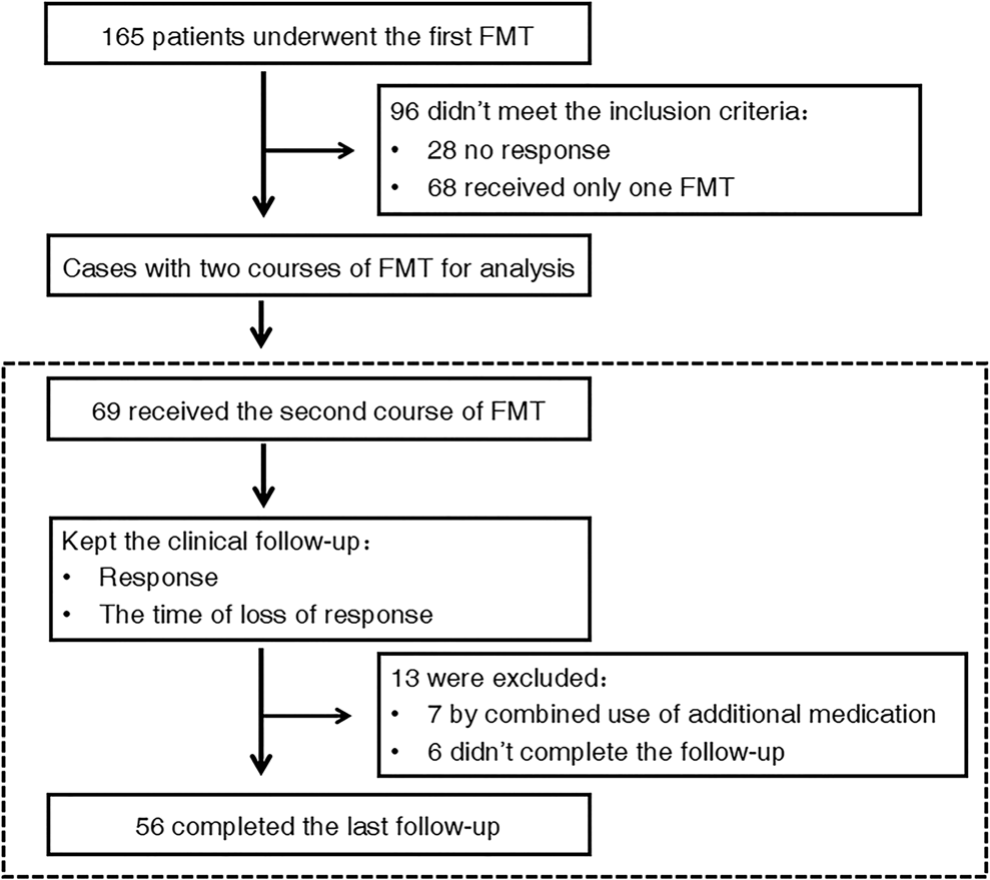
Flow chart of the study design. Loss of response referred to the flare after FMT-induced clinical response

This was a single-center pragmatic study. As shown in the flow chart in Fig. 1, FMT was administered to all eligible patients at baseline. Four weeks later, patients were assessed for the clinical response to the initial FMT. Patients who benefited from the first FMT were carefully followed for an extended period of time until the second course of FMT. The follow-up was performed at the third day, week 4, week 12, and later, every 3 months after the first FMT. Mesalazine 3.0 g daily was given to patients before the baseline and during the follow-up, and then the dose was reduced to 1.5–2.5 g daily according to our protocol if they were not allergic to this medication (Cui et al. 2015a).

Patients who were diagnosed as relapse of CD or had any indications of potential early active status of CD were regarded as the time for needing the second FMT for maintaining clinical response from the previous FMT. After the second FMT, follow-up was performed until the third FMT, or in April 2017. All subjects underwent endoscopy, routine complete blood count, erythrocyte sedimentation rate, and C-reactive protein testing prior to each FMT. The primary endpoint of this study was the patients’ clinical response maintaining time to the two FMTs. For maintenance time of the clinical response, it was defined as the time interval between the initial FMT and the disease recurrence or the potential early active status of CD. Another aim of the study was to explore the changes on gut microbiome and urine metabolome following the FMT.

### Outcome assessment and safety

Patients were assessed at the point of baseline, day 3, week 4, week 12, and every 3 months after each FMT. At surveillance point, the disease activity and severity were evaluated by physicians based on the HBI score or endoscopy and/or laboratory tests. The clinical efficacy was defined as follows: (1) partial improvement but patients themselves considered they benefited from the FMT (HBI > 4 and 1 ≤ HBI reduction ≤ 3), (2) clinical improvement (HBI > 4 and HBI reduction > 3), and (3) clinical remission (HBI ≤ 4). In our analysis, we classified those patients who achieved clinical improvement or remission from FMT as clinical responders. No response was defined as no clinical improvement or remission from FMT. The following events were considered no response: (1) switched to other therapies and (2) required surgical intervention for CD. Loss of response referred to the flare after FMT-induced clinical response. The patients who showed disease flare would receive another FMT therapy. Changes of medication regimen and serious adverse events throughout the whole follow-up of FMT were recorded. Here, the intensity and relationship of adverse events with FMT were defined based on the Common Terminology Criteria for Adverse Events (version 3.0) (Trotti et al. 2003). The intensity of adverse events was classified as mild, moderate, severe, or disabling. The relationship of adverse events with FMT was categorized as unrelated, possibly related, or related to FMT.

### Feces and urine sample collection

Stool and urine samples from the randomly selected nine patients were collected for microbiome and urine metabolome analysis at the baseline right before the first FMT (pre-first FMT), at 3 days after the first FMT (3D post-first FMT), and at the time point right before the second FMT (pre-second FMT). Stool samples from their respective donors at the first FMT were also collected for microbiome analysis.

### 16S ribosomal RNA gene sequencing and processing

Microbial DNA was extracted from stool samples. Bacterial 16S ribosomal RNA (rRNA) gene sequences were PCR amplified using bar-coded primers for the V4–V5 hypervariable region by the Phusion High-Fidelity PCR Master Mix with HF buffer (New England Biolabs, England). Products from each sample were mixed at equal molar ratios and then sequenced using the Illumina MiSeq platform (Illumina, Inc., San Diego, CA, USA), following standard Illumina sequencing protocols.

16S rRNA gene sequences were analyzed using a combination of software: mothur (version 1.33.3, http://www.mothur.org/), UPARSE (USearch version v8.1.1756, http://drive5.com/usearch/manual/uparse_pipeline.html), and R (version 3.2.3, https://www.r-project.org/). Operational taxonomic units (OTUs) were clustered at 97% similarity and filtered using the UPARSE pipeline. Unweighted UniFrac distances were calculated using mothur and visualized with principal coordinate analysis (PCoA) using R. Significance thresholds were adjusted to account for false discovery rate when making multiple comparisons using the Benjamini–Hochberg approach (Landy et al. 2015).

All 16S rRNA gene sequences have been deposited at the National Center for Biotechnology Information (NCBI) under the BioProject ID PRJNA428898 (https://www.ncbi.nlm.nih.gov/bioproject/PRJNA428898/).

### Urine nuclear magnetic resonance spectroscopic analysis

Urine samples were prepared according to a previously published protocol (Beckonert et al. 2007). Briefly, urine samples were thawed and then centrifuged to remove particulate matter. Two hundred microliters of phosphate buffer (pH = 7.4) containing 1 mM TSP (sodium (trimethylsilyl)[2,2,3,3-^2^H_4_]propionate) for internal standard was added to 400 μL of the supernatant. The mixture was centrifuged at 12,000*g* for 5 min at 4 °C, and then 550 μL of the supernatant was transferred into 5-mm nuclear magnetic resonance (NMR) tubes for analysis.

^1^H NMR spectra were acquired using nuclear Overhauser spectroscopy (NOESY) presaturation on a Bruker AV600 spectrometer (Bruker Co., Rheinstetten, Germany) at 298 K. Spectral raw data were determined by standard processing and calibration operations using Chenomx NMR Suite V.8.02 (Chenomx, Edmonton, Canada). Spectra were imported into the software and then were phased and baseline corrected. All the spectra were referenced to TSP (*δ* = 0.00 ppm). A “targeted profiling” approach (Stephens et al. 2013; Weljie et al. 2006) was applied where metabolites were identified and quantified using the 600 MHz library. Orthogonal partial least squares–discriminant analysis (OPLS-DA) was performed on log_10_-transformed metabolite abundance levels using SIMCA-P (version 13; Umetrics, Umeå, Sweden) that have been going through mean center and unit variance scaling, as a predictive model to explore the main effects in metabolite composition between the baseline (pre-first FMT) and after the first FMT (3D post-first FMT and pre-second FMT). The most discriminating variables were highlighted based on variable importance in the projection (VIP) with a value above 1. The significance of individual variables between different time points was further assessed using Wilcoxon signed-rank tests.

### Statistical analysis

Statistical analysis was performed using GraphPad Prism (version 5; GraphPad Software, San Diego, CA, USA). When the normality of the distribution of variables was acceptable, independent-samples *t* test and paired-samples *t* test were used. Otherwise, the Wilcoxon rank-sum test and Wilcoxon signed-rank test were used to analyze differences between groups.

## Results

### Patient characteristics

Sixty-nine patients with active CD who underwent FMT twice and benefited from the first FMT were included for analysis. The baseline characteristics for all 69 patients are shown in Table 1. 55.1% (38/69) of patients had moderate CD, and 44.9% (31/69) had severe CD. The average disease duration was 7.03 ± 5.48 years. Before the initial FMT, those patients had various medication regimens: 95.7% (66/69) were on 5-ASA, 53.6% (37/69) were on corticosteroids, 39.1% (27/69) were on immunomodulators, and 23.2% (16/69) were on anti-TNF agents.

**Table 1:** Baseline characteristics of patients

Variable	Results (*n* = 69)
Female sex, *n* (%)	31 (44.9)
Age, years, mean ± SD	35.8 ± 16.1
CD > 1 year, *n* (%)	63 (91.3)
Disease duration, years, mean ± SD	7.03 ± 5.48
Disease location, *n* (%)	
L1 = ileal	13 (18.8)
L2 = colonic	14 (20.3)
L3 = ileocolonic	42 (60.9)
L4 = upper GI tract involvement	6 (8.7)
Bowel surgery, *n* (%)	20 (30)
Anal surgery, *n* (%)	19 (27.5)
Medications before the first FMT, *n* (%)	
5-ASA	66 (95.7)
Corticosteroids	37 (53.6)
Immunomodulator	27 (39.1)
Anti-TNF therapy	16 (23.2)
Disease severity, *n* (%)	
Moderate	38 (55.1)
Severe	31 (44.9)

Values are presented as mean ± SD or number (%). The Montreal classification of CD was used to classify the disease extent

*FMT* fecal microbiota transplantation, *5-ASA* 5-aminosalicylic acid, *TNF* tumor necrosis factor, *CD* Crohn’s disease, *GI* gastrointestinal

### Clinical outcomes of the first two FMTs

**Fig. 2:**
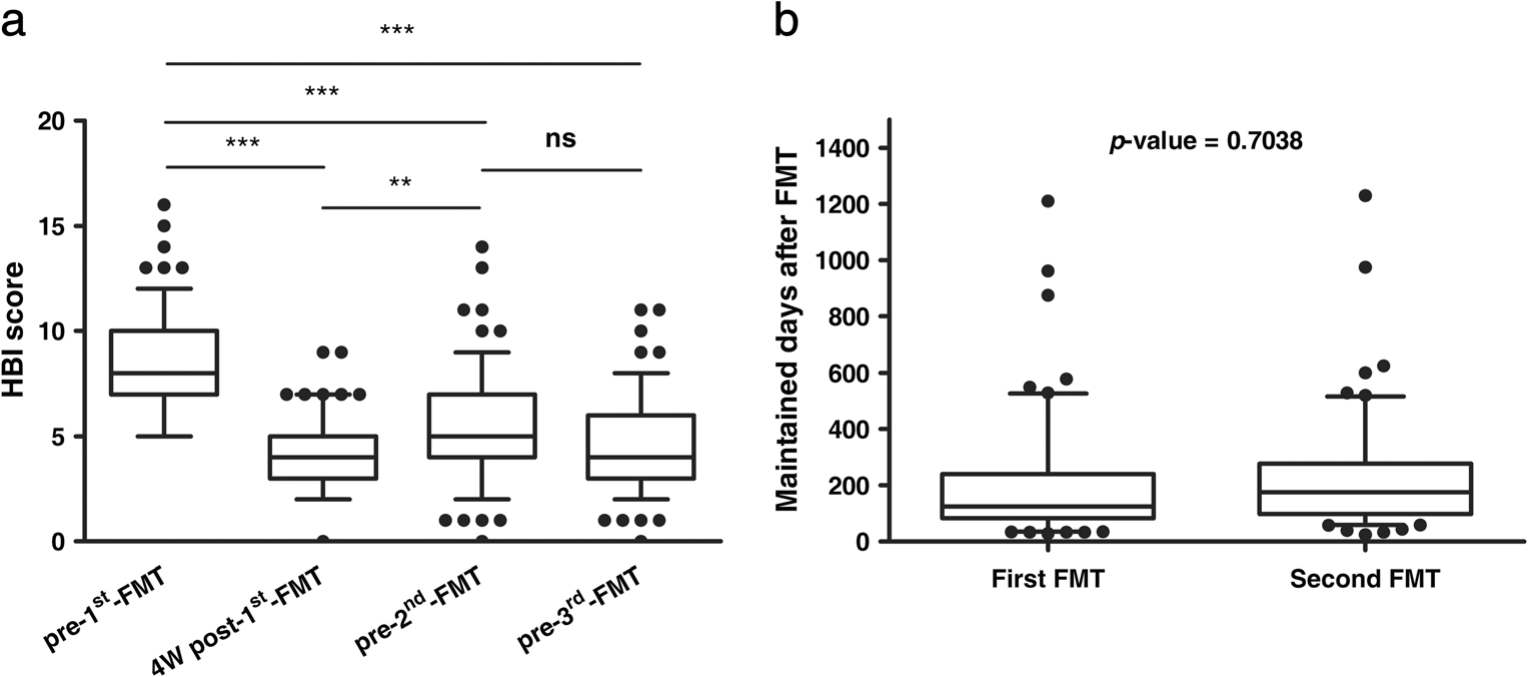
Harvey–Bradshaw Index (HBI) scores and clinical response maintaining time of all the patients (*n* = 69). **a** The change of HBI score after FMT. Compared with the baseline before the first FMT (pre-first FMT), the HBI score decreased significantly 4 weeks after the first FMT (4W post-first FMT) and right before the second FMT (pre-second FMT). Compared with the 4W post-first FMT HBI score, it increased significantly pre-second FMT. Significance levels: **p < 0.01, ***p < 0.001. **b** The comparison of patients’ clinical response maintaining time after the first and the second FMT (*p* > 0.05)

Four weeks after the first FMT, 63 patients achieved clinical response, of which 47 achieved clinical remission. In addition, 8.7% (6/69) of patients showed a partial improvement in CD-related symptoms. Right before those patients received the second FMT, 62.3% (43/69) of them still maintained a clinical response, among which 43.5% (30/69) still maintained clinical remission (Table 2). As shown in Fig. 2a, the HBI scores at 4 weeks after the first FMT significantly decreased than the scores prior to the first FMT (4.12 ± 1.69 vs. 8.51 ± 2.55, *p* < 0.001). And right before these patients received the second FMT, their HBI scores were still lower than the baseline scores before the first FMT (5.48 ± 2.92 vs. 8.51 ± 2.55, *p* < 0.001).

**Table 2:** Clinical outcome of the first FMT

Outcome	1st FMT (*n* = 69)		Pre-2nd FMT (*n* = 69)
	Week 0	Week 4	
Disease severity, *n* (%)			
No active and mild	–	–	30 (43.5)
Moderate	38 (55.1)	–	27 (39.1)
Severe	31 (44.9)	–	12 (17.4)
Clinical response to the FMT, *n* (%)			
Clinical improvement (HBI > 4, reduction > 3)	–	16 (23.2)	13 (18.8)
Clinical remission (HBI ≤ 4)	–	47 (68.1)	30 (43.5)
Partial response	–	6 (8.7)	–
Time from the first FMT to relapse, days, median (IQR)	–	–	125 (82.5–225.5)
HBI score	8.51 ± 2.55	4.12 ± 1.69***	5.48 ± 2.92***
ESR, mm/h	43.8 ± 29.3	–	43.6 ± 26.5
CRP, mg/L	29.2 ± 31.2	–	28 ± 29.3
High ESR, *n* (%)	50 (72.5)	–	57 (82.6)
High CRP, *n* (%)	44 (63.8)	–	47 (68.1)

All values are mean ± SD or number (%) unless otherwise stated

*FMT* fecal microbiota transplantation, *ESR* erythrocyte sedimentation rate (high ESR > 20 mm/h), *CRP* C-reactive protein (high CRP > 10 mg/L), *HBI* Harvey–Bradshaw index, *IQR* interquartile range

***p < 0.001

The median of the clinical response maintaining time for the first FMT was 125 days (IQR, 82.5–225.5). We set this time (125 days) as the benchmark to analyze the clinical response maintaining time of the second FMT. Seven patients, who obtained combined additional medication treatment (such as corticosteroids and immunomodulators) during the follow-up after the first FMT, were excluded in the analysis of the second FMT. 90.3% (56/62) of patients were followed up for more than 125 days after the second FMT. Among those patients, 64.3% (36/56) maintained clinical response to the second FMT for more than 125 days and 35.7% (20/56) maintained for less than 125 days. The median time of maintaining clinical response to the second FMT in those 56 patients was 176.5 days (IQR, 98.5–280). The box plot of patients’ clinical response maintaining time after FMT is shown in Fig. 2b. The patients’ clinical response maintaining time after the second FMT showed a higher median value than that after the first FMT, though the *p* value was not statistically significant. No severe adverse event related to the FMT was observed during and after the FMT procedure, as well as during the long-term follow-up.

### Gut microbiota dysbiosis in CD

**Fig. 3:**
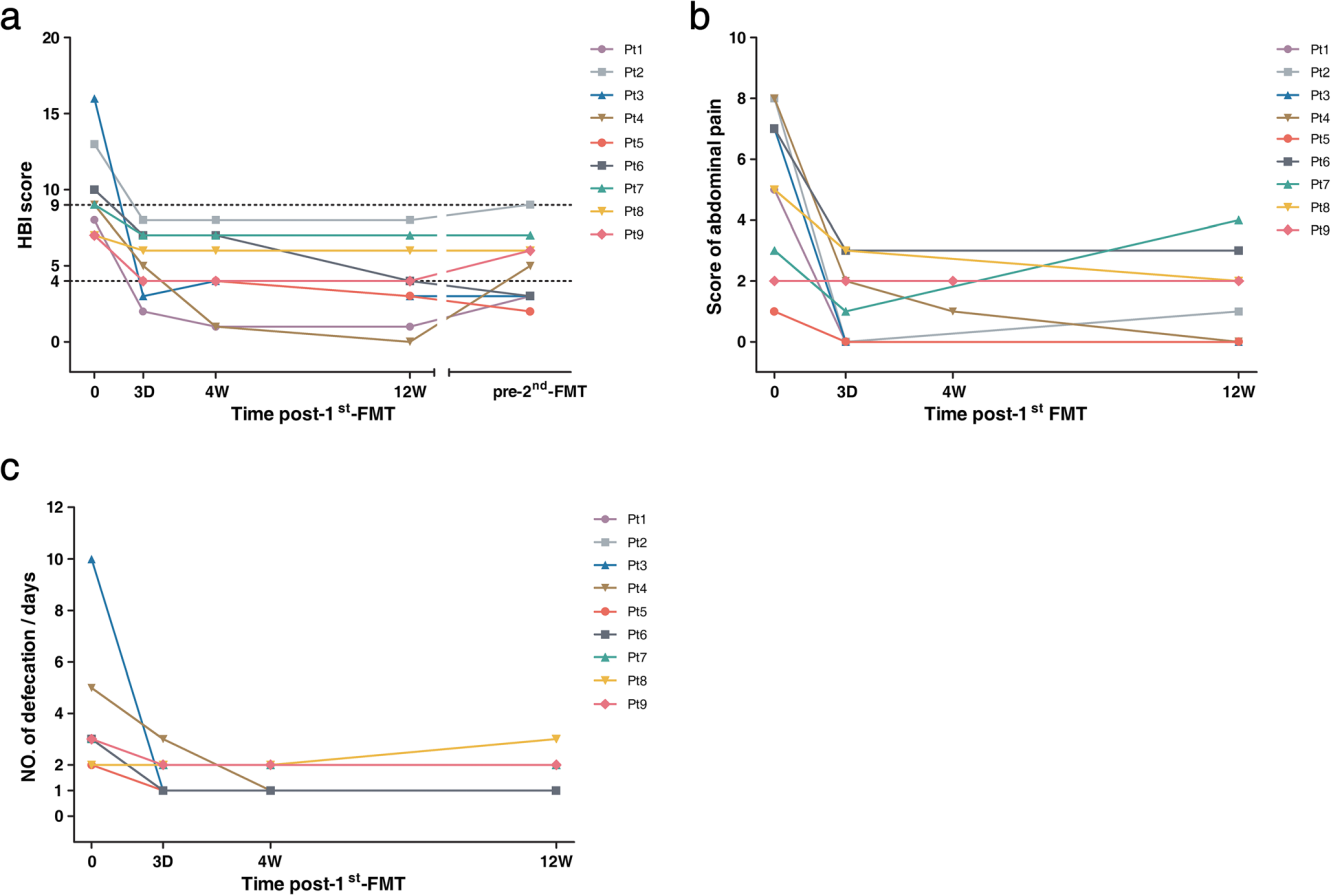
Clinical response to the first FMT in the selected nine CD patients. **a** HBI score at baseline; at 3 days, 4 weeks, and 12 weeks after the initial FMT; and at the time point right before the second FMT (pre-second FMT). **b** Abdominal pain scores at baseline and at 3 days, 4 weeks, and 12 weeks after the initial FMT. **c** Frequency of defecation at baseline and at and 3 days, 4 weeks, and 12 weeks after the initial FMT

Fecal samples from randomly selected nine CD patients and their respective healthy donors were collected to characterize the gut microbial compositions before and after the initial FMT. Note that among these nine patients, 66.7% (6/9) of them achieved clinical response at 4 weeks post-first FMT, while 44.4% (4/9) of them still maintained clinical response at the time point right before the second FMT, with the CD-related symptoms of abdominal pain and diarrhea sustaining relief (Fig. 3a–c).

**Fig. 4:**
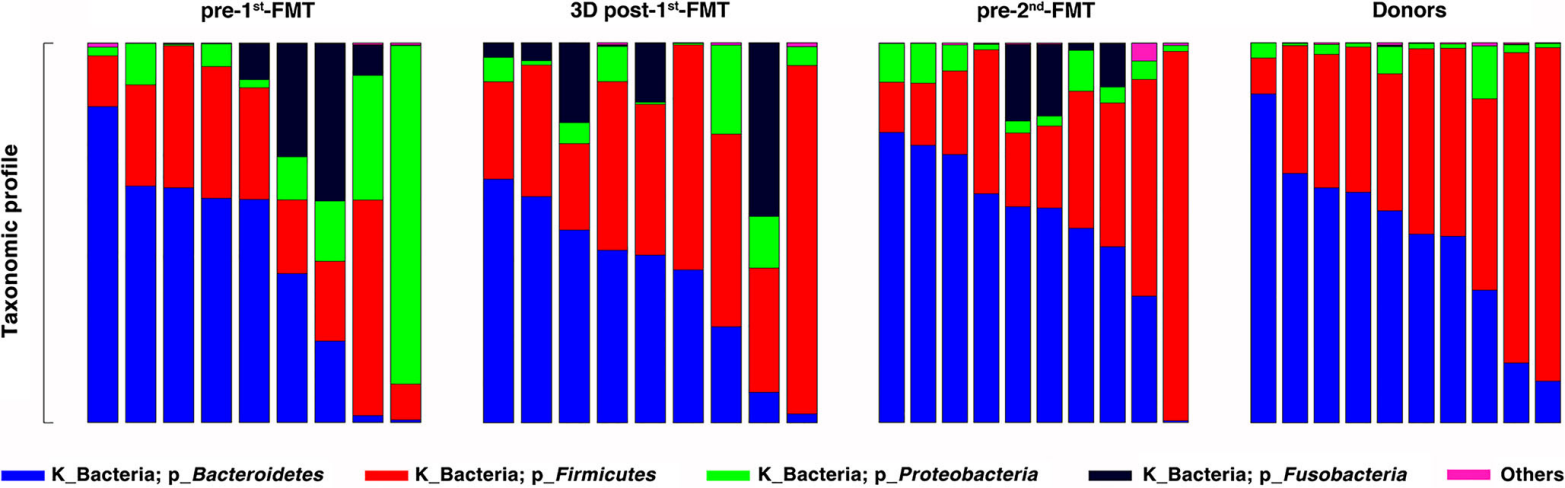
Microbial composition changes in the selected nine CD patients after the first FMT. **a** The microbial α-diversity (OTU richness) of donors and patients (*n* = 9) before the first FMT (pre-first FMT), 3 days after the first FMT (3D post-first FMT), and right before the second FMT (pre-second FMT). **b** The microbial α-diversity (Shannon diversity index) of donors and patients (*n* = 9) pre-first FMT, 3D post-first FMT, and pre-second FMT. The Wilcoxon rank-sum test was used to determine the significance between donors and patients, and the Wilcoxon matched-pairs signed-rank test was used between the samples before and after FMT. *p < 0.05, ***p < 0.001. **c** Principal coordinate analysis (PCoA) with unweighted UniFrac distance for donor and patient samples pre- and post-FMT. The gray lines showed the trajectory from donor’s to patient’s pre-first FMT, 3D post-first FMT, to pre-second FMT microbiome sample. *d*_1_ is the distance between donor’s and patient’s pre-first FMT microbiome sample. *d*_2_ is the distance between donor’s and patient’s 3D post-first FMT microbiome sample. *d*_3_ is the distance between donor’s and patient’s pre-second FMT microbiome sample. **d** *d*_1_ is always less than *d*_2_, implying that the taxa composition of the patient after the first FMT is closer to that of his/her donor. **e** *d*_3_ is typically less than *d*_1_, implying that the taxa compositions of most of the patients (with a few exceptions) before the second FMT are still close to those of their donors

The gut microbiome in CD patients displayed significantly smaller OTU richness than that of healthy donors (*p* = 0.0003, Fig. 4a). However, the Shannon index did not display significant difference between CD patients and their healthy donors, despite the fact that the Shannon index showed a decreasing trend (*p* = 0.077, Fig. 4b). PCoA based on the unweighted UniFrac distance revealed that the overall gut microbial compositions of active CD patients deviated from those of the healthy donors (Fig. 4c).

**Fig. 5:**
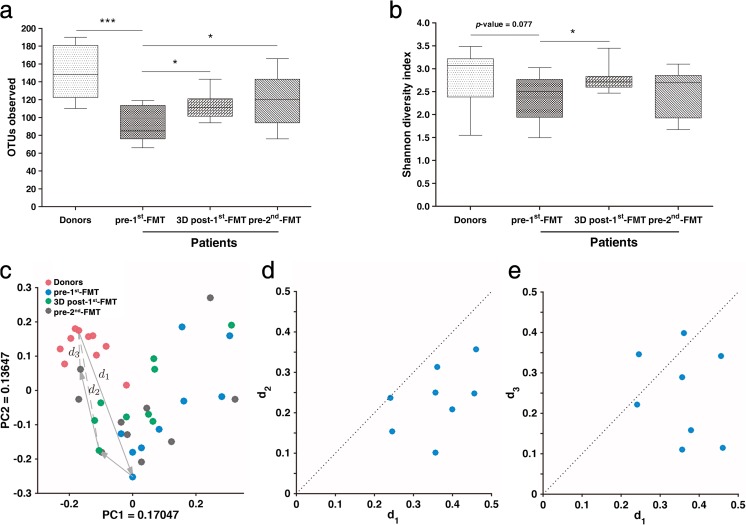
Gut microbial compositions of CD patients (*n* = 9) and donors (*n* = 9) at the phylum level. For patients, we sequenced their stool samples right before the first FMT (pre-first FMT), 3 days after the first FMT (3D post-first FMT), and right before the second FMT (pre-second FMT)

At the phylum level, differential abundance analysis showed that those pre-first FMT samples displayed a significant enrichment in *Fusobacteria* (*p* = 0.03) and a significant depletion of *Firmicutes* (*p* = 0.047), compared to the samples of healthy donors (Fig. 5, Supplemental Table S1). At the family level, we observed 11 bacterial taxa that displayed different abundance levels between CD patients and healthy donors (*p* < 0.05) (Supplemental Table S2). The relative abundance of bacterial families associated with CD such as *Fusobacteriaceae* (*p* = 0.030) and *Enterobacteriaceae* (*p* = 0.038) increased significantly in CD patients compared with that in healthy donors. At the genus level, several genera were observed to diminish significantly in CD patients compared with those in healthy individuals, including *Faecalibacterium*, *Lachnospira*, *Coprococcus*, *Dorea*, *Pseudomonas*, and *Anaerostipes* (*p* < 0.05). In addition, the genus *Ruminococcus* (*p* = 0.002) was significantly enriched in samples from CD patients compared with those from healthy donors (Supplemental Table S3).

### Changes of the gut microbiome following the first FMT

At the third day after the first FMT, the Shannon index and OTU richness of the gut microbiome from CD patients increased to significantly higher levels than the baseline, while before the second FMT, the OTU richness still increased significantly, but the Shannon index did not change dramatically, compared to the baseline (Fig. 4a, b). PCoA based on the unweighted UniFrac distance revealed a global difference in the overall microbial compositions between pre-FMT and post-FMT samples (Fig. 4c). Here, we defined three distances in PCoA: *d*_1_, the distance between donor’s and patient’s pre-first FMT microbiome samples; *d*_2_, the distance between donor’s and patient’s 3D post-first FMT microbiome samples; and *d*_3_, the distance between donor’s and patient’s pre-second FMT microbiome samples. We found that *d*_2_ was smaller than *d*_1_ for all the donor–patient pairs (Fig. 4d), and *d*_3_ was smaller than *d*_1_ for almost all the donor–patient pairs (Fig. 4e). These results indicate that FMT shifted the taxonomic composition of a patient’s gut microbiome toward that of his/her donor. And, this shift was effective even before the second FMT.

We compared several specific taxa previously associated with CD in subjects before and after FMT (Gevers et al. 2014). Among those families associated with CD, like *Fusobacteriaceae*, *Enterobacteriaceae*, and *Veillonellaceae*, no significant changes in the relative abundance were observed in the post-treatment group (Supplemental Table S4). Among the genera enriched in healthy controls, *Faecalibacterium* became more abundant in the post-treatment group (*p* < 0.05). Although several low-abundance genera had a nominal *p* < 0.05, very few differed significantly after adjusting significance threshold levels to account for false discovery rate (Supplemental Table S5).

**Fig. 6:**
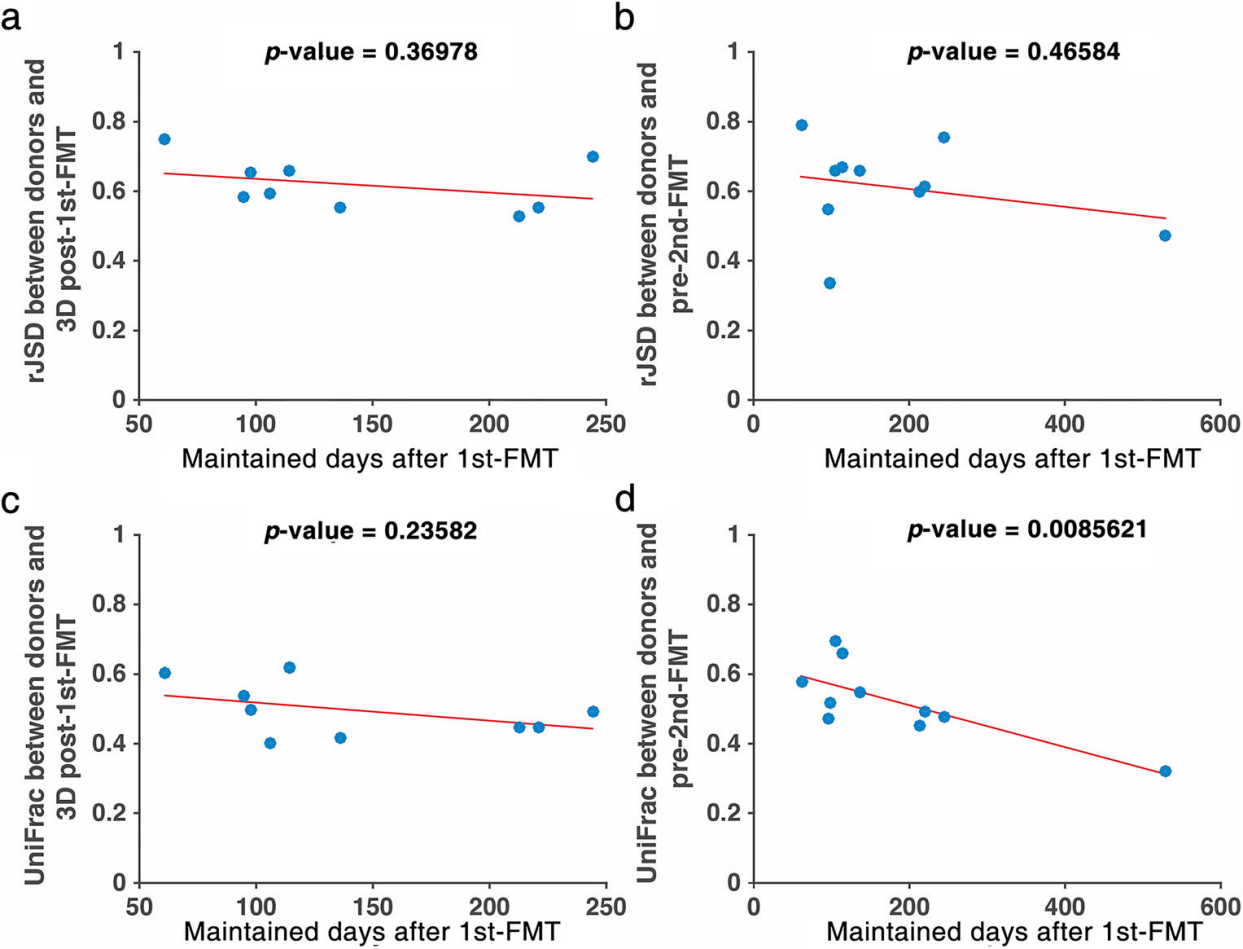
The relationship between microbial composition dissimilarity and clinical response maintaining time. Each dot represents a patient. For dissimilarity measures, we use **a**, **b** the root Jensen–Shannon divergence (rJSD) and **c**, **d** the unweighted UniFrac. **a**, **c** Dissimilarity between the donor’s and patient’s 3D post-first FMT microbiome samples. **b**, **d** Dissimilarity between the donor’s and patient’s pre-second FMT microbiome samples

We hypothesize that if the dissimilarity between donor’s and patient’s post-first FMT microbiome samples (3D post-first FMT or pre-second FMT) is small, the patient will maintain the clinical response to the first FMT for a long time. To test this hypothesis, we plotted the dissimilarity between microbiome samples at different time points as a function of the clinical response maintaining time (Fig. 6a–d). As shown in Fig. 6, we did observe a negative relationship. However, the *p* value for testing the null hypothesis was too large to convincingly conclude that the negative slope was statistically significant.

### Effects of the first FMT on urine metabolome

**Fig. 7:**
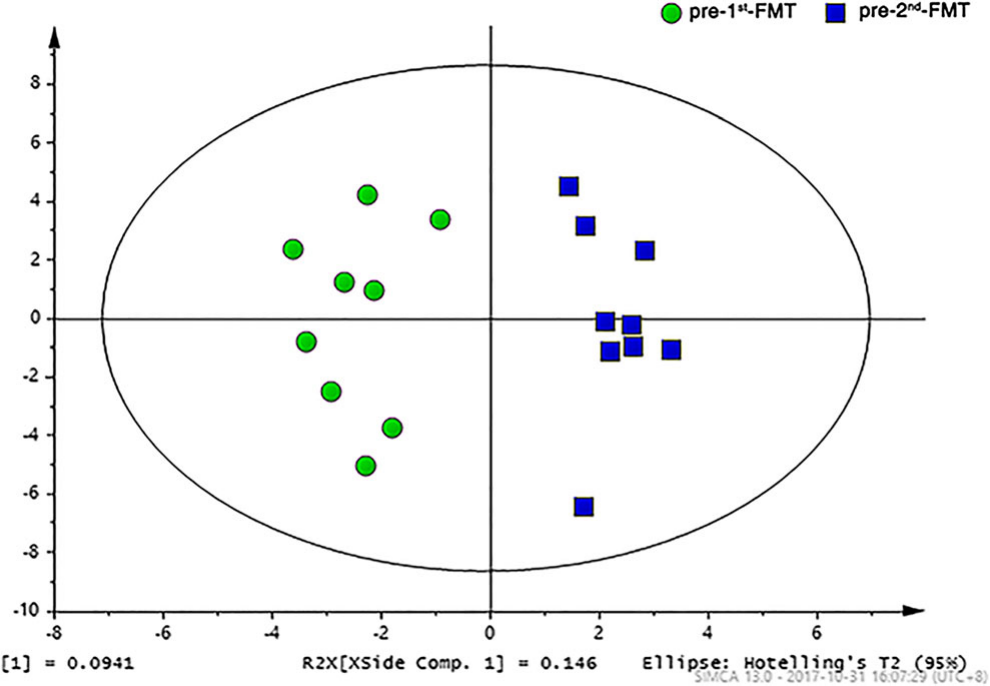
OPLS-DA score plot of ^1^H NMR profiles of urine samples obtained from the nine CD patients before the first FMT (pre-first FMT) and at right before the second FMT (pre-second FMT)

**Fig. 8:**
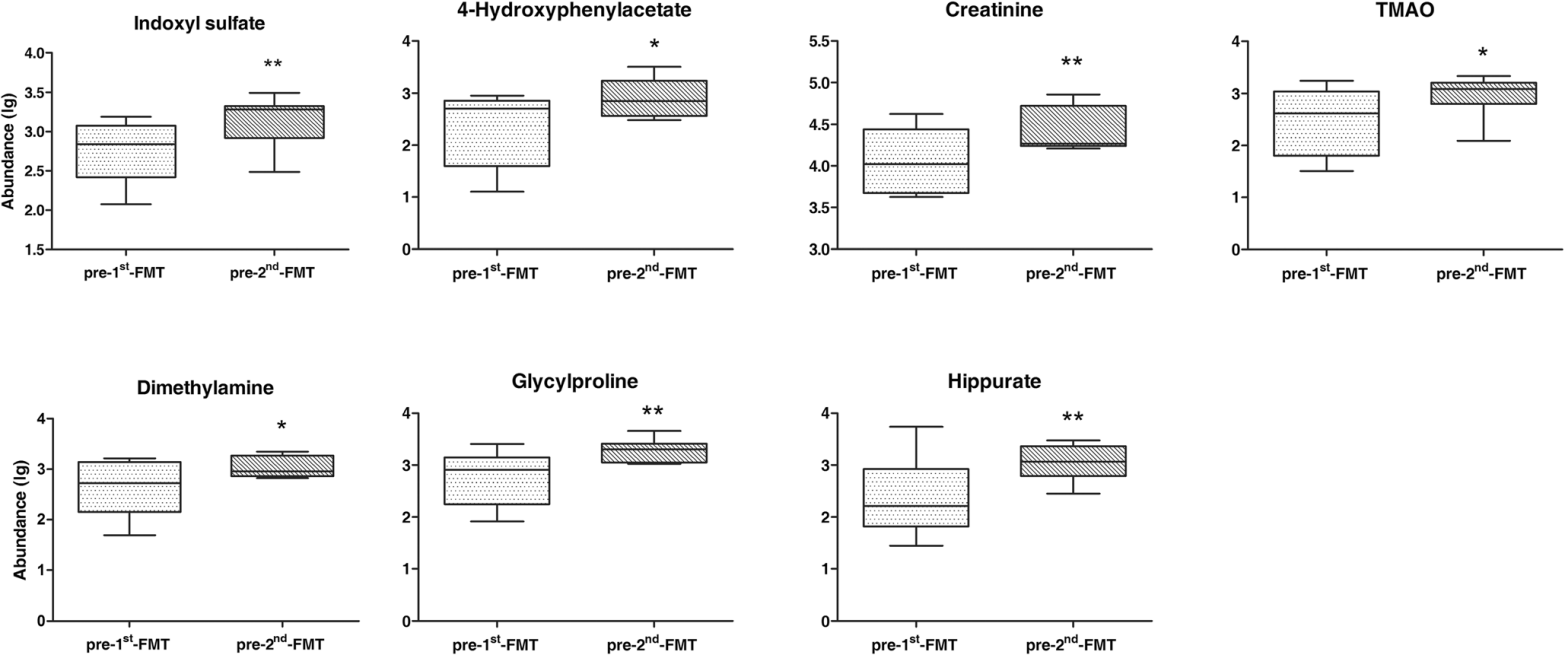
Urinary metabolite analysis. Changes of urinary metabolites between pre-first FMT and pre-second FMT (*n* = 9). Significance levels: *p < 0.05, **p < 0.01

To evaluate the effects of FMT on the host metabolism, we performed urinary metabolic profiling based on NMR spectroscopy. Sixty-nine metabolites were ultimately identified and quantified based on the comparison with the Chenomx metabolite database using the targeted profiling method. As shown in the OPLS-DA score plot (Fig. 7), a significant global metabolic difference in urine samples was achieved between the pre-first FMT and the pre-second FMT. Compared with that prior to the first FMT, seven metabolites including indoxyl sulfate, 4-hydroxyphenylacetate, creatinine, dimethylamine, glycylproline, hippurate, and trimethylamine oxide (TMAO) were elevated at the time right before the second FMT (Fig. 8).

## Discussion

Our recent survey of patients’ attitudes toward the use of FMT for CD demonstrated that 74.29% of patients who benefited from the initial FMT were willing to accept the second FMT (Xu et al. 2016). The current study aimed to evaluate the optimal timing to administer the second course of FMT for those patients. A total of 69 patients with active CD who benefited from the first FMT and underwent the second course of FMT were included in the final analysis.

Several pilot studies have shown that FMT could effectively induce clinical response in patients with active CD (Colman and Rubin 2014; Cui et al. 2015a; Goyal et al. 2018; Suskind et al. 2015; Vaughn et al. 2016). The clinical response rates of 86.7% and 66.7% (at 1 month and 6 months, respectively), 77.8% (7 of 9 patients), 57.9% (11 of 19 patients), 71% and 42% (at 1 month and 6 months, respectively) have been reported separately in four clinical trials (Cui et al. 2015a; Goyal et al. 2018; Suskind et al. 2015; Vaughn et al. 2016). However, how to maintain the long-term response of remodeling microbiota in CD based on FMT is another critical question.

In this study, we followed up the clinical response to the first FMT in the 69 patients with active CD. One month after the first FMT, 63 patients achieved clinical response and 47 of them achieved clinical remission. The other six achieved partial response after the first FMT, and their CD-related symptoms such as diarrhea, abdominal pain, bloody stool, and fever were improved. The median time of maintaining clinical response from the first FMT in all 69 patients was 125 days (4.17 months), indicating the time for second FMT should be less than 4 months. After the second FMT, 56 patients completed the follow-up, and 64.3% of them maintained clinical response over 125 days. Importantly, the median time of maintaining clinical response was 176.5 days (5.88 months). This indicates that serial FMTs might prolong the clinical efficacy of the previous FMT(s). This supported our previous instructions for patients with active CD that most of them should receive another FMT within 4 months after the previous FMT (Cui et al. 2015a; He et al. 2017). Three months after, the first FMT could be suggested in clinical practice for delivering the second course of FMT treatment on managing those patients with CD who had benefits from FMT. This strategy might be used in ulcerative colitis and other microbiota-related diseases which need long-term treatment using microbiota transplantation.

This study showed the microbiota dysbiosis in all the randomly selected nine CD patients before the first FMT. At the genus level, we found that the genus *Fusobacterium*, as a biomarker of IBD (Strauss et al. 2011), was significantly enriched in those CD patients (*p* = 0.03). Moreover, several protective bacterial genera displayed a significant depletion, such as *Faecalibacterium* (*p* = 0.001). Studies have shown that the decreased abundance of *Faecalibacterium prausnitzii* was significantly associated with the disease severity of CD (Fujimoto et al. 2013; Sokol et al. 2008). This depletion was restored after therapy, and *Faecalibacterium* became more abundant in the post-treatment group.

It has been reported that the gut microbiota composition of CD patients who experienced clinical response to FMT was closer to that of their donors, whereas for those non-responders, their gut microbiota composition did not have this change (Moayyedi et al. 2015; Vaughn et al. 2016). In our study, the gut microbiota composition of CD patients showed a high degree of similarity with their donors 3 days after the first FMT. Although some patients experienced a relapse of disease prior to the second FMT, their microbiota composition still showed a slight shift to their donors. The changing of microbiota after the first FMT indicates that the second FMT was necessary to restore the normal microbiota.

It has been well established that changes in the gut microbiota composition are associated with metabolic alterations in IBD (Ni et al. 2017). However, the impact of FMT on the metabolism in patients with active CD remains unclear. In our study, we observed no difference in urinary metabolic profiles between pre-FMT and 3 days post the first FMT. Surprisingly, at the time point right before the second FMT, the urinary metabolic profiles were significantly different from those before the first FMT. The metabolic changes were largely attributable to increased production of the following molecules: indoxyl sulfate, TMAO, dimethylamine, 4-hydroxyphenylacetate and hippurate, etc.

Indoxyl sulfate is a dietary protein metabolite and also a metabolite of the common amino acid tryptophan. Previous study has shown that urinary indoxyl sulfate could be used as an indirect marker for gut microbiome diversity, and its low concentration might reflect the disruption of the gut microbiota (Weber et al. 2015). In this study, we observed that FMT enhanced the production of urinary indoxyl sulfate and increased the diversity of gut microbiota. In addition, members of the families *Lachnospiraceae* and *Ruminococcaceae* were reported to be associated with a high level of urinary indoxyl sulfate (Weber et al. 2015). We also observed that the family *Ruminococcaceae* was enriched after the first FMT. It indicates that the urinary indoxyl sulfate might be useful to evaluate the effects of FMT on gut microbiota.

TMAO is produced by gastrointestinal anaerobes through the digestion of dietary phosphatidylcholine and carnitine in a microbial–mammalian co-metabolic pathway and might serve as a biomarker for IBD (Wilson et al. 2015). In this study, the increased urinary concentration of TMAO might be due to the successful colonization of anaerobic bacteria in the gut after FMT. Dimethylamine is highly abundant in human urine, and its main sources have been reported to include TMAO and asymmetric dimethylarginine (ADMA) (Tsikas et al. 2007). The present results showed a significant increase in urinary dimethylamine after FMT, which was likely due to the high abundance of TMAO.

In addition, for 4-hydroxyphenylacetate and hippurate which belong to the phenolic, benzoyl, and phenyl derivatives, it has been reported that their urinary concentrations were associated with the gut microbiota composition and activity (Nicholson et al. 2012). Interestingly, hippurate has been linked to the presence of *Clostridia* class in the gut (Storr et al. 2013), and the relative abundance of *Clostridia* was tightly associated with the level of intestinal inflammation (Kolho et al. 2015). Furthermore, previous studies have shown that downregulation of the hippurate in CD patients was associated with the altered gut microbial metabolism (Dawiskiba et al. 2014; Williams et al. 2010).

There were some limitations in the present study. The duration of the clinical response to the first two FMTs was primarily based on patients’ self-report, and the disease activity was not confirmed by objective indicators such as calprotectin and lactoferrin. The measurement of gut microbiota and urine metabolites was only performed in limited patients. It was difficult to exclude the effects of diet on the gut microbiota and urinary metabolome in our study. Larger sample size of population should be more powerful to identify the microbial and metabolic signatures.

In conclusion, this study demonstrated that the median time for maintaining the clinical response from FMT in CD patients was about 4 months. The present results indicated that less than 4 months (3 months as suggested from practical view) after the initial FMT could be suggested as the second course of FMT for maintaining clinical response from FMT. The supportive results showed that FMT could affect the host–microbial metabolism and contribute to the significant urinary metabolic changes in patients with active CD.

## Electronic supplementary material

ESM 1 (PDF 367 kb)
