# Supplementary material for: Timing for the second fecal microbiota transplantation to maintain the long-term benefit from the first treatment for Crohn’s disease
Source: Appl Microbiol Biotechnol. 2018 Oct 24;103(1):349–60. doi: 10.1007/s00253-018-9447-x (PMC6311185; doi:10.1007/s00253-018-9447-x)
Supplement: Supplementary file 1 — (PDF 367 kb) [file 253_2018_9447_MOESM1_ESM.pdf]

**Supplementary material**

**Applied Microbiology and Biotechnology**

# Timing for the second fecal microbiota transplantation to maintain the long-term benefit from the first treatment for Crohn's disease

Pan Li[1,2] , Ting Zhang[1,2], Yandong Xiao[3,4], Liang Tian[3], Bota Cui[1,2], Guozhong Ji[1,2], Yang-Yu Liu[3,5], Faming Zhang[1,2]

[1] Medical Center for Digestive Diseases, the Second Affiliated Hospital of Nanjing Medical University, Nanjing 210011, China.

[2] Key Lab of Holistic Integrative Enterology, Nanjing Medical University, Nanjing 210011, China.

[3] Channing Division of Network Medicine, Brigham and Women's Hospital and Harvard Medical School, Boston, Massachusetts 02115, United States.

[4] Science and Technology on Information Systems Engineering Laboratory, National University of Defense Technology, Changsha 410073, China.

[5] Center for Cancer Systems Biology, Dana-Farber Cancer Institute, Boston, Massachusetts 02115, United States.

Pan Li and Ting Zhang contributed equally to this study.

**Corresponding author**:

Faming Zhang, MD, PhD

Professor of Medicine

Director of Intestinal Diseases

The Second Affiliated Hospital of Nanjing Medical University

Tel: +86-25-58509883

E-mail address: fzhang@njmu.edu.cn

ORCID: 0000-0003-4157-1144

Yang-Yu Liu, PhD

Assistant Professor at Harvard Medical School

Associate Scientist at Brigham and Women’s Hospital

Tel: +1-617-525-2714

E-mail address: yyl@channing.harvard.edu

ORCID: 0000-0003-4184-5379

**Supplemental Table Legends**

**Supplemental Table S1** Gut microbial compositions in CD patients and donors at the phylum level.

**Supplemental Table S2** Gut microbial compositions in CD patients and donors at the family level.

**Supplemental Table S3** Gut microbial compositions in CD patients and donors at the genus level.

**Supplemental Table S4** Gut microbial compositions in CD patients before and after FMT at the family level.

**Supplemental Table S5** Gut microbial compositions in CD patients before and after FMT at the genus level.

**Supplemental Table S1** Gut microbial compositions in CD patients and donors at the phylum level.

| Phylum | Donor (%) | Patient pre-1st-FMT (%) | *p*-value |
|---|---|---|---|
| *Cyanobacteria* | 0.0283 ±0.0371 | 0.0197 ±0.0591 | 0.023 |
| *Fusobacteria* | 0.0811 ±0.1506 | 9.97 ±15.33 | 0.030 |
| *Firmicutes* | 48.33 ±24.11 | 27.59 ±14.35 | 0.047 |

**Supplemental Table S2** Gut microbial compositions in CD patients and donors at the family level.

| Family | Donor (%) | Patient pre-1st-FMT (%) | *p*-value | FDR |
|---|---|---|---|---|
| *Ruminococcaceae* | 23.62 ±11.36 | 3.43 ±2.91 | 0.0013 | 0.0825 |
| *Clostridiaceae* | 2.27 ±3.44 | 0.46 ±0.69 | 0.0243 | 0.2665 |
| *Enterobacteriaceae* | 1.86 ±3.26 | 15.34 ±24.65 | 0.0380 | 0.2687 |
| *Coriobacteriaceae* | 0.12 ±0.19 | 0.02 ±0.03 | 0.0054 | 0.1427 |
| *Fusobacteriaceae* | 0.08 ±0.15 | 9.95 ±15.34 | 0.0302 | 0.2665 |
| *Bifidobacteriaceae* | 0.06 ±0.06 | 0.12 ±0.35 | 0.0095 | 0.2010 |
| *Bacteroidales_S24-7* | 0.04 ±0.04 | 0.007±0.006 | 0.015 | 0.2265 |
| *Comamonadaceae* | 0.009 ±0.007 | 0.002 ±0.005 | 0.0175 | 0.2325 |
| *Sphingomonadaceae* | 0.008 ±0.008 | 0.002 ±0.007 | 0.0395 | 0.2791 |
| *Pseudomonadaceae* | 0.006 ±0.005 | 0.0005 ±0.002 | 0.0030 | 0.1047 |
| *Carnobacteriaceae* | 0.001 ±0.002 | 0.01 ±0.02 | 0.0330 | 0.2665 |

**Supplemental Table S3** Gut microbial compositions in CD patients and donors at the genus level.

| Genus | Donor (%) | Patient pre-1st-FMT (%) | *p*-value | FDR |
|---|---|---|---|---|
| *Ruminococcus* | 0.330 ±0.342 | 2.115 ±5.521 | 0.0023 | 0.0752 |
| *Anaerostipes* | 0.378 ±0.731 | 0.058 ±0.170 | 0.0124 | 0.2091 |
| *Bifidobacterium* | 0.060 ±0.055 | 0.117 ±0.350 | 0.0095 | 0.1924 |
| *Blautia* | 1.282 ±1.040 | 0.875 ±1.980 | 0.0469 | 0.3751 |
| *Collinsella* | 0.093 ±0.174 | 0.004 ±0.009 | 0.0376 | 0.3751 |
| *Coprococcus* | 0.281 ±0.352 | 0.007 ±0.008 | 0.0007 | 0.0632 |
| *Dorea* | 0.746 ±0.652 | 0.431 ±1.080 | 0.0092 | 0.1924 |
| *Enterobacter* | 0.001 ±0.002 | 1.564 ±4.664 | 0.0418 | 0.3751 |
| *Faecalibacterium* | 12.590 ±12.067 | 0.763 ±1.407 | 0.0013 | 0.0632 |
| *Fusobacterium* | 0.080 ±0.150 | 9.947 ±15.322 | 0.0302 | 0.3751 |
| *Granulicatella* | 0.001 ±0.002 | 0.010 ±0.013 | 0.0330 | 0.3751 |
| *Lachnospira* | 1.508 ±1.670 | 0.006 ±0.010 | 0.0003 | 0.0632 |
| *Pseudomonas* | 0.006 ±0.005 | 0.001 ±0.002 | 0.0030 | 0.0752 |
| *Roseburia* | 0.694 ±0.474 | 1.064 ±2.447 | 0.0458 | 0.3751 |
| *Firmicutes_SMB53* | 0.530 ±0.850 | 0.031 ±0.065 | 0.0029 | 0.0752 |
| *Sphingomonas* | 0.008 ±0.008 | 0.002 ±0.007 | 0.0395 | 0.3751 |

**Supplemental Table S4** Gut microbial compositions in CD patients before and after FMT at the family level.

| Family | Pre-1st-FMT (%) | 3D post-1st-FMT (%) | [a] *p*-value | FDR | Pre-2nd-FMT (%) | [b] *p*-value | FDR |
|---|---|---|---|---|---|---|---|
| *Bacteroidaceae* | 36.70 ±26.89 | 33.45 ±19.07 | 0.8886 | 1.000 | 47.34 ±17.95 | 0.4413 | 0.938 |
| *Enterobacteriaceae* | 15.34 ±24.65 | 6.53 ±8.25 | 0.4008 | 0.8603 | 3.45 ±4.02 | 0.3139 | 0.7272 |
| *Veillonellaceae* | 12.55 ±12.28 | 13.97 ±9.39 | 0.7794 | 1.000 | 9.97 ±10.60 | 0.6784 | 1.000 |
| *Fusobacteriaceae* | 9.95 ±15.34 | 11.36 ±15.82 | 0.8886 | 1.000 | 5.83 ±8.60 | 0.6784 | 1.000 |
| *Ruminococcaceae* | 3.43 ±2.91 | 8.13 ±8.52 | 0.1614 | 0.7481 | 11.45 ±15.79 | 0.2604 | 0.7272 |
| *Porphyromonadaceae* | 2.07 ±1.95 | 5.19 ±5.52 | 0.208 | 0.7481 | 4.21 ±3.32 | 0.0209 | 0.7272 |
| *Clostridiaceae* | 0.46 ±0.69 | 0.60 ±0.69 | 0.4008 | 0.8603 | 0.43 ±0.30 | 0.9528 | 1.000 |
| *Aeromonadaceae* | 0.2 ±0.56 | 0.007 ±0.018 | 0.0431 | 0.7481 | 0.018 ±0.033 | 0.735 | 1.000 |
| *Bifidobacteriaceae* | 0.12 ±0.35 | 0.008 ±0.01 | 0.0679 | 0.4696 | 0.06 ±0.14 | 0.7150 | 1.000 |
| *Gemellaceae* | 0.043 ±0.072 | 0.079 ±0.18 | 0.753 | 1.000 | 0.0019 ±0.0023 | 0.0280 | 0.7272 |
| *Desulfovibrionaceae* | 0.042 ±0.068 | 0.11 ±0.10 | 0.0180 | 0.7481 | 0.054 ±0.074 | 0.161 | 0.7272 |
| *Bacteroidales_S24-7* | 0.007 ±0.006 | 0.01 ±0.03 | 0.3454 | 0.7982 | 0.27 ±0.58 | 0.0663 | 0.7272 |

[a] *p*-value shows differences in relative abundance between 3D post-1st-FMT/pre-1st-FMT.

[b] *p*-value shows differences in relative abundance between pre-2nd-FMT/pre-1st-FMT.

**Supplemental Table S5** Gut microbial compositions in CD patients before and after FMT at the genus level.

| Genus | Pre-1st-FMT (%) | 3D post-1st-FMT (%) | [a] *p*-value | FDR | Pre-2nd-FMT (%) | [b] *p*-value | FDR |
|---|---|---|---|---|---|---|---|
| *Ruminococcus* | 2.115 ± 5.521 | 3.016 ± 3.967 | 0.0357 | 0.5101 | 1.841 ± 3.068 | 0.4413 | 0.6995 |
| *Bilophila* | 0.042 ± 0.068 | 0.088 ± 0.078 | 0.0180 | 0.5101 | 0.029 ± 0.059 | 0.7794 | 0.8295 |
| *Faecalibacterium* | 0.763 ± 1.407 | 3.807 ± 7.291 | 0.2076 | 0.5101 | 3.265 ± 4.147 | 0.0382 | 0.5562 |
| *Gemella* | 0.022 ± 0.042 | 0.054 ± 0.128 | 0.7532 | 0.8175 | 0.001 ± 0.002 | 0.0277 | 0.5562 |
| *Holdemania* | 0.004 ± 0.008 | 0.179 ± 0.294 | 0.0464 | 0.5101 | 0.037 ± 0.055 | 0.0357 | 0.5562 |
| *Parabacteroides* | 2.069 ± 1.950 | 5.194 ± 5.521 | 0.2076 | 0.5101 | 4.214 ± 3.321 | 0.0209 | 0.5562 |

[a] *p*-value shows differences in relative abundance between 3D post-1st-FMT/pre-1st-FMT.

[b] *p*-value shows differences in relative abundance between pre-2nd-FMT/pre-1st-FMT.
